# Immunohistochemical diagnosis of human infectious diseases: a review

**DOI:** 10.1186/s13000-022-01197-5

**Published:** 2022-01-30

**Authors:** Hamadou Oumarou Hama, Gérard Aboudharam, Rémi Barbieri, Hubert Lepidi, Michel Drancourt

**Affiliations:** 1 IHU Méditerranée Infection, Marseille, France; 2 Aix-Marseille-Univ., IRD, MEPHI, IHU Méditerranée Infection, Marseille, France; 3 Aix-Marseille-Univ., Ecole de Médecine Dentaire, Marseille, France; 4 Laboratoire d’Histologie, Faculté de Médecine, Université de la Méditerranée, Marseille, France

**Keywords:** Immunohistochemistry, infectious diseases, past infections, diagnosis

## Abstract

**Background:**

Immunohistochemistry (IHC) using monoclonal and polyclonal antibodies is a useful diagnostic method for detecting pathogen antigens in fixed tissues, complementing the direct diagnosis of infectious diseases by PCR and culture on fresh tissues. It was first implemented in a seminal publication by Albert Coons in 1941.

**Main body:**

Of 14,198 publications retrieved from the PubMed, Google, Google Scholar and Science Direct databases up to December 2021, 230 were selected for a review of IHC techniques, protocols and results. The methodological evolutions of IHC and its application to the diagnosis of infectious diseases, more specifically lice-borne diseases, sexually transmitted diseases and skin infections, were critically examined. A total of 59 different pathogens have been detected once in 22 different tissues and organs; and yet non-cultured, fastidious and intracellular pathogens accounted for the vast majority of pathogens detected by IHC. Auto-IHC, incorporating patient serum as the primary antibody, applied to diseased heart valves surgically collected from blood culture-negative endocarditis patients, detected unidentified Gram-positive cocci and microorganisms which were subsequently identified as *Coxiella burnetii*, *Bartonella quintana*, *Bartonella henselae* and *Tropheryma whipplei*. The application of IHC to ancient tissues dated between the ends of the Ptolemaic period to over 70 years ago, have also contributed to paleomicrobiology diagnoses.

**Conclusion:**

IHC plays an important role in diagnostic of infectious diseases in tissue samples. Paleo-auto-IHC derived from auto-IHC, is under development for detecting non-identified pathogens from ancient specimens.

**Supplementary Information:**

The online version contains supplementary material available at 10.1186/s13000-022-01197-5.

## Introduction

Diagnosis of infectious diseases is fundamentally based on the isolation by culture of the causative pathogen and isolation by culture of pathogens remains the gold standard method for the laboratory diagnosis of infectious diseases [1]. The choice of methods used for isolation by culture depends in part on the nature of the sample, the microorganism to be identified, and the conditions under which the samples are processed and stored. However in some circumstances, rate of isolation of microorganisms from cultures in tissue biopsies may be low and serological diagnosis can be difficult; as illustrated for an example, for the search for the tuberculosis agent *Mycobacterium tuberculosis* in biopsy specimens such as diseased lymph node biopsies [2, 3]. In recent decades immunohistochemistry has become an indispensable alternative for pathologists due to two major technical advances and the use of specific antibodies against various antigens. The application of monoclonal or polyclonal antibodies to viral, bacterial or fungal antigens in order to characterise infectious agents in immunohistochemistry is now routinely used in the diagnosis of many infectious diseases [4–6]. However, like any other diagnostic method, immunohistochemistry requires quality assurance, reproducibility and sensitivity in order to detect a targeted infectious agent. Thus, to avoid variations in immunostaining and to maintain the immunoreactivity of certain antigens, several factors need to be taken into account, mainly tissue fixation, tissue processing and antigen retrieval [7–9]. Furthermore, as the antigens had been recovered from ancient paraffin blocks and mummified bodies, the preservation of antigenic epitopes dating back at least a century has been demonstrated by immunohistochemical staining, despite the degradation of certain antigenic determinants in ancient tissues [6, 10]. In this study, we review immunohistochemistry techniques and protocols as applied to the diagnosis of infectious diseases, including past infections in the context of paleomicrobiology.

## Bibliographical methods

**Fig. 1:**
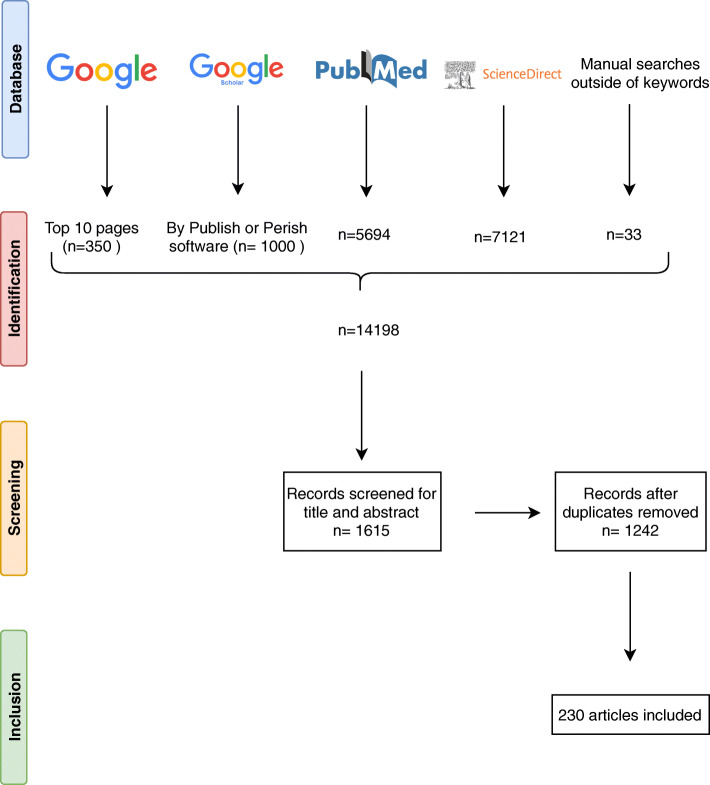
Summary of the process in a flow diagram

We searched the literature for relevant articles in the PubMed, ScienceDirect, Google Scholar and Google databases. The pre-selection of articles based on titles and abstracts was complete by July 2020 (and was updated in December 2021), and the keywords used for the search were “immunohistochemistry, immunohistochemical, human infections, diagnosis” (Fig. 1). In addition, for the Google Scholar database, “Publish or Perish” software was used to pre-select the 1,000 best articles associated with the keywords and, for the Google database, the top 10 pages (n=350) were pre-selected. Manual searches were performed for articles outside the keywords and also in the reference lists of the pre-selected articles to find other relevant sources. Experimental and animal studies were excluded from our selection and the final selection of papers was based on immunohistochemical methods and the crucial contribution that immunohistochemistry can make to the diagnosis of infectious diseases in humans.

## The historical development of immunohistochemistry and automation

**Fig. 2:**
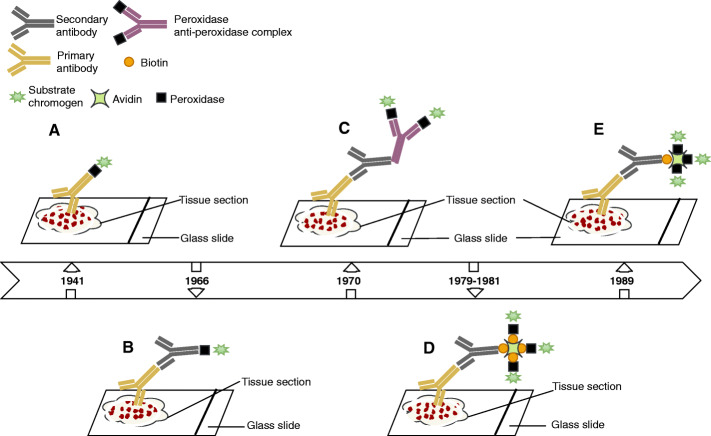
Schematic representation of immunohistochemical methods. **A**: direct method, **B**: indirect method, **C**: PAP complex procedure, **D**: ABC procedure, **E**: LAB procedure

Immunohistochemical staining is derived from immunofluorescence, and dates back to 1941, when Coons and colleagues demonstrated that tissues stained with fluorescein-conjugated antibodies became fluorescent specifically under ultraviolet light [11] (Fig. 2). Although immunofluorescence has been widely used in immunology for the diagnosis of diseases, this method has a number of limitations related to the use of fluorescein namely the natural autofluorescence of the tissues which masks the specific fluorescence, the lack of stability of the preparations, and the use of ultraviolet microscopes which are expensive and difficult to use. In order to overcome some of these shortcomings, alternative immunostaining methods have been developed. One alternative to fluorescent antibodies involves staining the tissue by methods of labelling antibodies with enzymes (Fig. 2) that react with non-fluorescent chromogenic substrates [12]. Preparations stained with antibodies and conjugated to enzymes such as peroxidase are permanent and can be observed with ordinary light microscopes, enabling the simultaneous observation of antigen localisation and tissue morphology. The use of unlabelled antibodies (peroxidase-antiperoxidase technique: **PAP**) (Fig. 2) to identify antigens by immunohistochemistry increased sensitivity [13]. A subsequent development involved using alkaline phosphatase for double immunoenzyme labelling (alkaline phosphatase and peroxidase), which was capable of detecting two antigens within the same cell [14]. However, the application of a secondary labelled antibody to biotin followed by the addition of the avidin-biotin peroxidase complex (**ABC**) (Fig. 2) proved to be much more sensitive than the unlabelled antibody method [15, 16]. In addition, the peroxidase-labelled avidin-biotin (**LAB**) method offered even greater sensitivity than previous immunohistochemical methods [17], due to the fact that the peroxidase enzyme is directly covalently bound to the avidin molecule (Fig. 2). Epitope retrieval through enzymatic digestion was another productive step forward in the use of immunohistochemistry in formalin-fixed and paraffin-embedded tissues [18].

Due to these advances in immunohistochemistry, tissue antigen analysis became the undisputed gold standard for pathologists and anatomists for clinical diagnoses or experimental research based on tissue biopsies. The use of monoclonal antibodies made it possible to detect an increasing number use of specific markers against the antigens represented in tissues and against infectious agents from formalin-fixed paraffin blocks, and has led to increased pressure on industry. The increase in the number of tests each year in research and particularly in hospital laboratories resulted in technical problems related to the workforce, reagent stability, reproducibility, temperature variation of the different steps of an immunoassay, and a reliability crisis [19]. A viable automated immunohistochemistry system to detect tissue antigens was first invented and developed by Brigati and colleagues as an alternative to manual methods. This fully automated system (from de-paraffining to nuclear staining) overcame the restrictions on volume and discrete analysis by using the capillary action with the help of standardised software and a computer. The retrieval antigen from formalin-fixed, paraffin-embedded tissues by heat pre-treatment was a crucial step forward in the maintenance and success of immunohistochemistry [20]. This important publication resulted in the detection of antigens by immunohistochemical staining on formalin-fixed archival tissues dating back at least a century [21–23]. In the years following the automation of immunohistochemistry, considerable improvements were observed in the development of the automatons i.e., reagent cost management, reagent application, slide labelling, traceability of operations and analytical flexibility [24–26].

## Immunohistochemistry procedures

The examination of hematoxylin and eosin-stained tissue sections is the first step in the diagnosis of any infectious disease from tissue samples before any other staining in histopathology [6]. The principal immunohistochemical methods are direct, indirect, PAP, alkaline phosphatase and avidin-biotin techniques [17, 27], which use antigen-antibody complexes to locate cellular antigens in paraffin sections, frozen tissues, post-mortem tissues and cell preparations.

Currently, the labelled streptavidin-biotin method is the most widely used routine laboratory method for diagnosing diseases. It has the same principle as the LAB method, i.e., the primary antibody against the antigen of interest is bound to the enzyme-labelled streptavidin (the enzyme is covalently bound to the streptavidin) through the biotin present on the secondary antibody and revealed with a chromogenic substrate [26, 28].

### Tissue preparation

Immunohistochemical staining involves different steps in the technical treatment of tissues that can influence the correct interpretation of the results. These are the procedures of fixation, dehydration and paraffin embedding. Regarding fixation, two types of fixatives are commonly used, namely reticulation fixatives (e.g. formalin) and coagulation fixatives (alcohol solutions such as ethanol, methanol and acetone) [29]. Formalin is a standard fixative that has been used for decades. The increased needs of modern histopathological laboratories considered standard fixation modes too long for the preservation of immunoreactivity [30], as somewhere between 12 and 36 hours is required for complete fixation with 10% neutral buffered formalin, for example [31]. The fixation time in formalin can play an important role in obtaining a reliable result in immunohistochemistry, such as in the determination of oestrogen receptors in breast cancer, which was at least six to eight hours [32]. A large part of variations observed in immunohistochemistry were caused by the very short fixation time due to a variable mixture of cross-linking and coagulation fixation during the tissue dehydration step by ethanol [9]. In tissue diagnostics, the fixation time of formaldehyde, which is generally used at 10% in a neutral phosphate buffer, must be minimised for the effective application of the immunohistochemical staining [33, 34]. Prolonged fixation can produce a progressive loss of certain tissue antigens used in diagnosis, such as lymphocyte antigens which are lost after three days’ fixation [33]. An alternative to neutral buffered formalin and a prolonged fixation time is formalin zinc, which allows better morphological conservation and preserves immunoreactivity [30]. Coagulation fixatives are dehydrating agents, which replace water in the tissue environment and thus destabilise the hydrophobic binding of proteins. This disruption of tertiary proteins structure mainly causes the loss of functions and the insolubility of proteins [35]. This protein denaturation phenomenon does not affect all antigens and depends on the alcohol concentration, the presence of organic and inorganic substances, the pH and the fixation temperature.

However, regardless of the choice of fixation mode, fixation alone does not cause the loss of antigen recognition but rather occurs after a combination of fixation, tissue treatment and paraffin embedding. Studying the effects of different fixatives under the same conditions of treatment and embedding, found that dehydrating fixatives such as ethanol 95% or methanol 100% and alcoholic formaldehyde best preserved the immunorecognition of p53 and large spectrum keratins, while the best fixers for TGFα and p185 ^erbB-2^ were cross-linking fixers (unbuffered 10% formalin and unbuffered zinc formalin) [36]. After fixation, tissue treatment and embedding the tissue was dehydrated in ethanol and then the ethanol was removed by a clarification agent such as xylene, which infiltrates the tissue [37]. After the hot paraffin dissolves in the xylene, the xylene evaporates and the incorporation of paraffin into the tissue is complete once the tissue has cooled. This process is carried out using an automaton within 12 hours. Thus, using a heating mould containing molten paraffin, a paraffin block is constructed in which the tissue infiltrated by the paraffin is trapped. This paraffin infiltration into the tissue allows 4-μm sections to be cut in order to visualise the structure, but this may also depend on the nature of the sample and the position of the tissue. The loss of reactivity of some antigens due to dehydration, paraffin embedding, paraffin wax temperature and during fixation of sections on microscopic slides has previously been discussed [29, 37]. It should be noted that the most important problem related to the variation in immunohistochemical staining is inadequate tissue dehydration. This can be avoided by renewing solutions weekly and applying consistently [9].

#### Antigen retrieval

Antigen retrieval by enzymatic digestion from tissue sections requires only two hours of trypsin treatment to reveal certain immunoreactive sites of the antigens [18]. However, some antigens are not revealed by trypsin digestion, with the exception of cytokeratins and desmin [38]. This method has been replaced by microwave heating of formalin-fixed tissue sections. Subsequent methods consist in microwave-heating paraffin tissue sections at up to 100°C in the presence of metallic solutions [20]. This pre-treatment improves immunohistochemical procedures due to a considerable improvement in antigen recovery, including in long-term formalin-fixed tissues. Antigens of interest are then revealed using chromogenic substrate after several incubation steps with antibodies, as presented in the Supplementary Material.

#### Automation

The routine use of immunohistochemistry in diagnostic laboratories had resulted in its automation. The staining procedure remains the same as in manual immunohistochemistry, but it is controlled from a programmed computer. The advantages and disadvantages of automated immunohistochemistry vary according to the different commercially available immunohistochemistry platforms. The choice of these platforms depends on the needs of the laboratory, according to the capacity of the different systems. Unlike the automated immunohistochemical method, the manual method provides extensive staff knowledge with almost infinite flexibility in the choice of reagents and recovery methods as well as the ability to introduce certain technical variations when optimising the protocol. However, the automation of immunohistochemistry has made immunohistochemical testing reproducible, fast and accurate with processing of multiple slides at the same time, high and consistent labelling quality, analytical flexibility, standardisation of critical steps, time saving, low cost, and remote operation with a user-friendly interface and biosafety [24–26, 39]. Furthermore, despite the development of automated machines that require periodic monitoring and maintenance, the steps of tissue fixation, paraffin block construction and tissue cutting remain a human task.

## Immunohistochemical detection of bacterial pathogens

### *Bartonella quintana* (*B. quintana*)

*B. quintana* is a Gram-negative bacillus responsible for trench fever, bacteraemia, endocarditis, bacillary angiomatosis and chronic lymphadenopathy [40]. Trench fever was discovered during the First World War, linked to poor living conditions and lice infestation (Brouqui *et al.*, 1999; Maurin and Raoult, 1996). *B. quintana* endocarditis is a fatal infection of global importance the current approach to which involves the Duke diagnostic criteria [41]. Immunohistochemistry successfully identified *B. quintana* in the heart valves of patients with blood culture-negative infective endocarditis [42]; making histopathological examination of heart valves mandatory for the diagnosis of infective endocarditis [43].

### *Yersinia pestis* (*Y. pestis*)

**Fig. 3:**
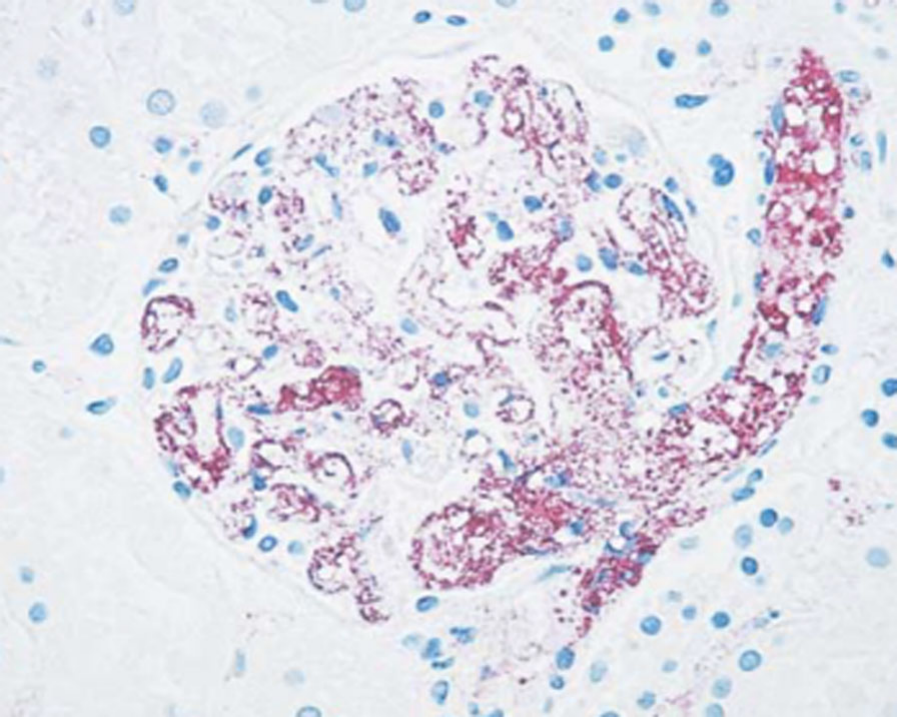
Immunohistochemical stain of a kidney sample demonstrating granular antigen staining delineating the blood vessels in the glomerulus (anti fraction 1 *Y. pestis* antibody, ×100)

*Y. pestis* is a Gram-negative bacterium responsible for plague, a zoonosis transmitted by ectoparasites including fleas and possibly lice resulting in lymph node infection (so-called bubo), which spreads as septicaemia and pneumonia [44]. Microscopy, culture and polymerase chain reaction (PCR) are the diagnostic methods used for the direct detection of *Y. pestis* [45, 46]. However, culture and direct immunofluorescence require specimens (sputum, blood or aspirations from lymph nodes) which are often difficult to obtain and dangerous to handle, hence the usefulness of immunohistochemical testing to detect *Y. pestis* in formalin-fixed tissues in order to preserve the morphological features and to minimise handling of dangerous specimens [47]. Indeed *Y. pestis* was identified intact by an immunohistochemical test inside monocytes and granular antigen staining in blood vessels (Fig. 3). Immunohistochemistry was also used to confirm pulmonary plague during a plague epidemic in Ecuador [48].

### *Treponema pallidum* (*T. pallidum*)

**Fig. 4:**
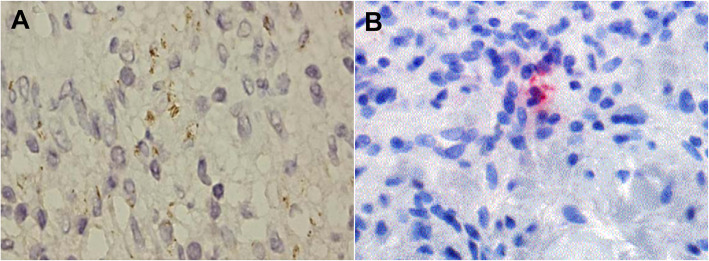
**A** Immunohistochemistry for syphilis showing spirochetes in the cytoplasm of histiocytes (1000x, oil immersion). **B.** Immunohistochemistry showing *C. trachomatis* particles in lesional tissue

Treponematoses such as yaws, bejel and syphilis are infections caused by *T. pallidum* involving the different subspecies *T. pallidum pertenue*, *T. pallidum endemicum*, and *T. pallidum*, respectively [49]. Treponemal infections, more particularly syphilis, have a worldwide impact on human health but remain indistinguishable through routine morphology and serology. Treponemes are fastidious organisms whose culture and maintenance on artificial media remains difficult, despite new techniques in microbiology [50, 51]. Silver staining of spirochetes in tissue sections was the usual method for identifying *T. pallidum*, but the immunohistochemistry technique using a monoclonal antibody was found to be more sensitive and appropriate to avoid marked background staining and to facilitate the observation of spirochetes in secondary syphilis tissue sections [52]. Immunohistochemistry is more sensitive and specific than silver staining for detecting *T. pallidum* in tissue sections [53–55], but should be interpreted with care, as immunostaining with the *T. pallidum* antibody (Biocare) also stains some acid-fast bacilli and *Helicobacter pylori* [56]. The efficacy of immunohistochemistry for *T. pallidum* detection has been proven in biopsies of unsuspected oral lesions [57] and in biopsies of skin lesions [58], and its utility has been proven in the diagnosis of syphilitic chancre [59, 60] papulonodular secondary syphilis [61], malignant syphilis [62] and erythema multiforme caused by *T. pallidum* [63]. The combination of immunohistochemistry and PCR has been shown to be effective for the diagnosis of secondary syphilis [64] and confirmation of syphilitic orchitis in an HIV-infected young man with a false-negative Venereal Disease Research Laboratory test and *T. pallidum* agglutination test (Fig. 4A) [65].

### *Chlamydia trachomatis* (*C. trachomatis*)

*C. trachomatis*, an obligate intracellular bacterium, is the most common sexually-transmitted infection in the world [66, 67]. The detection of *C. trachomatis* infection is based on PCR and serology [66]. An immunohistochemical investigation incorporating a monoclonal antibody reactive against *C. trachomatis* D/K and L2 serovars (Acris Antibodies catalogue number AM00660PU-N) detected *C. trachomatis* in skin lesions in two cases of lymphogranuloma venereum (Fig. 4B) [68]. Despite the greater sensitivity of DNA detection methods (PCR and ligase chain reaction) compared to immunohistochemistry for the detection of *C. trachomatis* from fixed biopsies, the application of immunohistochemistry remains essential for pathological diagnosis [69, 70].

### Other bacteria

Several microorganisms are responsible for infective endocarditis, most commonly Gram-positive cocci (such as Staphylococci, Streptococci and Enterococci), *B. quintana* (already described above), *Bartonella henselae* (the agent of cat scratch disease) *Coxiella burnetii* (the agent of Q fever) and *Tropheryma whipplei* (the agent of Whipple’s disease) [42, 71–73]. However, when it comes to the antimicrobial treatment of patients and certain fastidious or non-cultivable microorganisms, identification of these bacteria by blood culture may be difficult [74, 75]. Therefore, pathological examination of valve tissue sections remains the reference technique for the diagnosis of infective endocarditis, despite the advances in molecular techniques and serological tests [43, 76]. Accordingly, immunohistochemistry is routinely used for the detection of *Bartonella* spp., *T. whipplei* and *C. burnetii*, *S. aureus*, Group A Streptococci, and for the pathological diagnosis of infectious diseases using specific antibodies [42, 72, 73, 77–79]. A related method incorporating the patient’s own serum as the primary antibody, known as auto-immunohistochemistry, successfully detected bacteria in the heart valves of blood-culture negative endocarditis patients [71].

Despite cross-reactivity between the *Mycobacterium tuberculosis* polyclonal antibody and normal eosinophil granules [80], IHC can be hugely useful in identifying cutaneous mycobacteriosis [81–84] and certain bacterial infections associated with *Clostridium s*pecies [79], *Borrelia burgdorferi* [85], *Helicobacter pylori* [86–90], *Rickettsia rickettsii* [91–93], *Chlamydia pneumoniae* [94, 95], *Orientia tsutsugamushi* [96–99], *Neisseria meningitidis* [100], *Brachyspira* species [101], and *Burkholderia pseudomallei* [102].

## Immunohistochemical detection of viral pathogens

### Human Herpesviruses (HHV)

HHV are intracellular pathogens that have been classified into three subfamilies and have been divided into eight types based on their biological properties: *Alphaherpesviridae* (herpes simplex virus type 1, herpes simplex virus type 2, and varicella-zoster virus) have a neurocutaneous tropism. They can replicate and spread rapidly to many cell systems causing extensive cell lysis and can establish latent infection primarily in neurosensory ganglia [103].

**Fig. 5:**
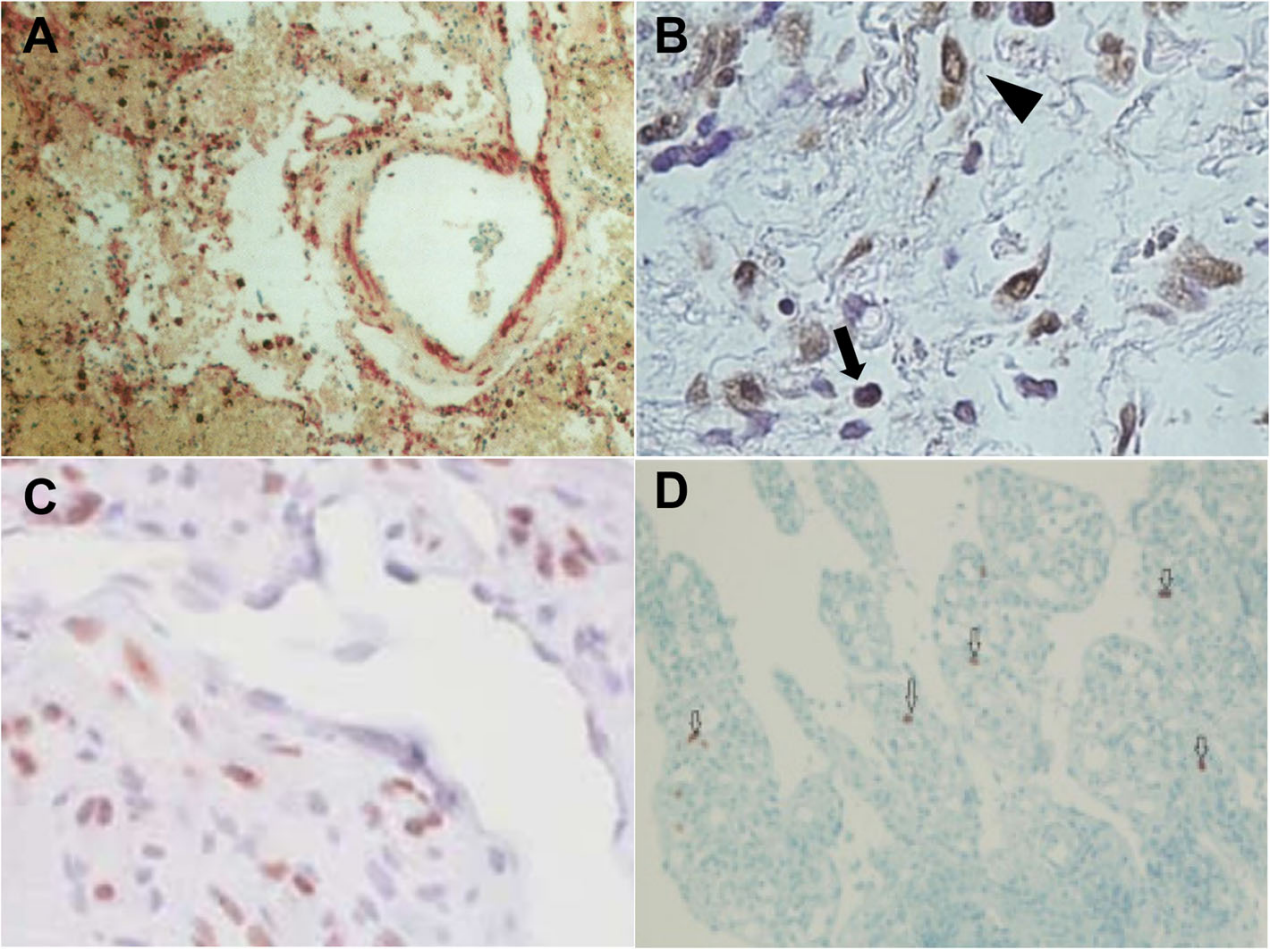
**A** HSVI immunoreactivity in the lung (IBD4 monoclonal antibody). **B**. LMP-1 immunohistochemistry showing positive staining in B cells (arrows) and plasma cells (arrow heads). Scale bar = 50μm. **C**. Immunohistochemical staining for HHV-8 LNA-1 in cutaneous patch/plaque Kaposi sarcoma. **D**. Immunohistochemistry of CMV showing nuclear positive cells in ileal tissue. x20

Herpes simplex virus (HSV) infections are among the most common diseases in humans, largely causing subclinical infections and usually manifesting in ulcerative lesions at the site of infection [104, 105]. Immunohistochemistry using polyclonal or monoclonal antibodies has been shown to be a sensitive and specific technique for diagnosing HSV infections when characteristic intranuclear inclusions or multinucleated cells are absent in biopsy specimens [2]. Discriminating HSV-1 from HSV-2 by immunohistochemistry is achieved using monoclonal antibodies and, in some cases, immunohistochemistry has been preferable to *in situ* hybridisation, as in the case of necrotic lesions, in which immunohistochemical detection has revealed the presence of HSV, while *in situ* hybridisation was negative (Fig. 5A) [106, 107].

Primary infection with VZV results in varicella and a resurgence in zoster [104]. The histological features of HSV can be observed in patients with varicella-zoster and it has been possible to identify varicella-zoster in immunohistochemistry by using a monoclonal antibody directed against the VZV envelope glycoprotein gpI [108] and glycoprotein E [109].

Immunohistochemistry has been very useful for the diagnosis of HSV-1/2 and VZV skin infections from fixed biopsies [110–114].

*Gammaherpesviridae* (Epstein-Barr virus and HHV-8) are the only human herpesviruses with an established oncogenic potential, causing a variety of lymphoproliferative and neoplastic disorders [103]. The viruses of this subfamily replicate *in vitro* in lymphoblastoid cells, sometimes in epithelioid and fibroblastic cells, and are specific for T or B lymphocytes in which they can induce latent or lytic infections [103, 104].

Epstein-Barr virus (EBV) was discovered in 1964 by electron microscopy from a Burkitt’s lymphoma cell line, a B-cell derived tumour. EBV is ubiquitous, infecting more than 90% of adults worldwide by establishing a lifelong persistent infection of B cells characterised by excretion of the virus in saliva, with most primary infections occurring sub-clinically in childhood [103, 104]. Immunohistochemistry identified EBV latent membrane protein 1 using tissue sections in apical and periapical periodontitis lesions (Fig. 5B) [115, 116], in patients with nasopharyngeal carcinoma [117], in immunocompetent adult [118], and in Hodgkin's disease and non-Hodgkin's lymphoma [119–121].

Human herpesvirus type 8 (HHV8), also known as Kaposi's sarcoma-associated herpes virus, is a recent member of the herpes family, discovered in 1994 in a Kaposi’s sarcoma (KS) skin lesion in an AIDS patient, thus establishing a link between HHV-8 infection and the emergence of KS [122]. Histological diagnosis of HHV8 is problematic because of its broad morphologic spectrum and the limitation of various benign and malignant vascular neoplasms [123]. In such cases, immunohistochemistry using an anti-HHV-8 LNA-1 antibody has proven to be a reliable marker with high sensitivity and specificity for the diagnosis of KS (Fig. 5C) [124–129].

*Betaherpesviridae* (cytomegalovirus, human herpesvirus type 6, and human herpesvirus type 7) replicate in a limited number of cellular systems and grow slowly in cell culture. These viruses cause little cell lysis and can establish latent infections in secretory glands, the lymphoreticular system, the kidneys and certain other tissues [103].

The histological diagnosis of cytomegalovirus (CMV) in fixed tissues has proven to be the “gold standard” for the identification of viral inclusions and other cytopathic effects, despite the lack of sensitivity in some cases, such as the difficulty in interpreting atypical cytopathic features with reactive or degenerative changes [130–132]. Combined immunohistochemistry and *in situ* hybridisation tests compare favourably with culture, allowing for faster diagnosis than immunofluorescence technique or culture for early anti-CMV therapy [133]. In addition, immunohistochemistry is much more sensitive than cytomorphology and *in situ* hybridisation for the detection of CMV in smears fixed from bronchoalveolar lavage samples [134]. David and colleagues recommended automated *in situ* hybridisation or immunohistochemistry for CMV detection in formalin-fixed, paraffin-embedded tissues [135]. Immunohistochemistry using monoclonal antibodies has detected CMV antigens from fixed tissues in septic patients [136], in placental biopsies [137, 138], in the brain after liver transplantation [139], in pulmonary and gastrointestinal tissues (Fig. 5D) [140–143], in apical periodontitis lesions [116], in tumours and in peripheral blood [144], in patients with steroid refractory ulcerative colitis [145] and in one patient with ischemic colitis [146].

HHV6 was isolated and characterised for the first time in 1986 by Salahuddin and collaborators [147], from peripheral blood mononuclear cells in six patients with lymphoproliferative disorders, two of whom were co-infected with human immunodeficiency virus (HIV). HHV-6 infection usually occurs at the age of two years and is the aetiological cause of exanthema subitum, also known as roseola infantum.

Immunohistochemical analysis with a monoclonal antibody against HHV-6 envelope glycoprotein (gp60/110 kDa) in two immunocompetent individuals demonstrated the presence of numerous viral inclusions in paracortical areas of the lymph nodes, and that atypical cells were positive for CD3 and CD4 [148, 149]. Furthermore, immunohistochemical staining has demonstrated the common presence of HHV6 in renal allografts [150], in bone marrow transplant recipients [151], in the central nervous system of neurological pathology patients and healthy people [152, 153] and in one infant infected with HIV and encephalitis [154]. HHV-6 antigens were also detected in B- and T-cell lymphoma tissues by immunohistochemistry using the polyclonal HHV6 antibody [155].

Another closely related virus, HHV-7, is also known to be the causative agent of exanthem subitum [103, 156, 157]. In 1990, HHV-7 was detected by Frenkel and colleagues in a culture of activated CD4+ T cells from a healthy individual [158].

Although rare, the detection of HHV-7 by immunohistochemistry has been successful in brain autopsy samples from unspecified encephalopathy cases [153] and in fixed normal tissues, suggesting that HHV-7 causes a persistent infection rather than a true latent one [153, 159].

### Human Immunodeficiency Virus (HIV)

HIV is a retrovirus belonging to the Retroviridae family, and is responsible for Acquired Immunodeficiency Syndrome (AIDS) [103]. Biological diagnosis is based primarily on serology and PCR.

**Fig. 6:**
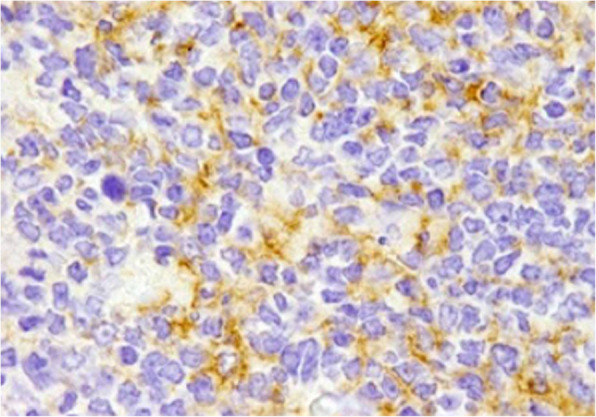
Positive immunohistochemical staining for HIV-1 p24 antigen in follicular centres and dendritic cells (original magnification ×400)

The immunohistochemical technique is rapid and efficient in identifying HIV antigens in fixed surgical and autopsy specimens [160]. Immunohistochemistry using an anti-p24 monoclonal antibody has proven useful for detecting HIV in lymph node biopsies of patients with unexplained follicular hyperplasia [161], in duodenal and rectal biopsies of AIDS patients [162], in cervical biopsy samples of HIV-infected women [163], and in a parotid gland biopsy from a rare case of HIV-associated benign lymphoepithelial cysts (Fig. 6) [164]. This approach has been recommended for the diagnosis of recent infections in which anti-p24 antibodies are not yet detectable in the serum or if morphological or clinical features suggest HIV infection [161, 165].

### Other viruses

Immunohistochemistry has been of great value for the laboratory diagnosis of Ebola haemorrhagic fever, especially during the epidemic in the Democratic Republic of Congo in 1995 [166, 167], and also for the elucidation of the pathogenesis of influenza A (H1N1) and B virus infection from fixed autopsy specimens [168–170]. Immunohistochemistry has been one of the main methods used to better understand the pathogenesis of Middle East respiratory syndrome coronavirus (MERS-CoV) in humans in fixed tissues obtained from the first autopsy performed on a fatal MERS-CoV case in the world [171]. The use of immunohistochemistry in fixed tissues has been essential as a diagnostic method and for providing further evidence of enterovirus involvement in myocarditis or dilated cardiomyopathy [172–176]. Immunohistochemistry has been useful in the diagnosis and clinical management of chronic hepatitis C [177–179], in the diagnosis of yellow fever [180, 181], hepatitis E [182], rabies [183–185], severe acute respiratory syndrome (SARS) [186], in the identification and pathogenesis of SARS-CoV-2 [187–193], in the identification of infections associated with Zika virus [194], West Nile virus [195–197], adenoviruses [198–200], hantavirus [201, 202], and in the diagnosis of other cutaneous viral infections by parvoviruses, poxviruses, paramyxoviridae [203].

## Immunohistochemical detection of fungal pathogens

The great majority of fungi are easily identified by hematoxylin-eosin staining alone or in combination with special stains such as Gomori methenamine silver stain and Schiff periodic acid stain which are used in routine histopathology [204, 205]. However, the morphological diagnosis of deep mycoses, ideally done by culture, can be time-consuming and often has to be made from tissue sections for smears, when cultures are not available [206]. Immunohistochemistry can be an accurate and efficient diagnostic tool for a number of important mycoses in humans and for the localisation of typical or atypical fungal elements in lesions from fixed tissue sections. Immunohistochemistry has been used for the diagnosis of pythiosis [207], fungal sinusitis [208], aspergillosis [209–211], sporotrichosis [212], and for the identification of *Candida albicans* [213], and *Cryptococcus neoformans* var. gattii [214].

## Immunohistochemical detection of protozoal pathogens

The diagnosis of protozoal infections is difficult because of the distortion of the morphology of the parasite due to the necrosis or autolysis of tissues or in cases of unusual presentation of the disease, hence the usefulness of immunohistochemistry (Arnold*et al.*, 1997).

Despite the higher sensitivity of PCR compared to immunohistochemistry for the diagnosis of leishmaniasis, the use of both tests together has been shown to be more effective for routine diagnosis [215, 216]. Immunohistochemistry significantly improves the diagnosis of cutaneous leishmaniasis in histological sections and allows the patient's immune response to be evaluated [217–219]. In addition, immunohistochemistry has been useful in the diagnosis of toxoplasmosis [220–222], echinococcosis [223, 224], malaria [225] and for the detection of *Trypanosoma cruzi* [226, 227].

## Paleoimmunohistochemical detection of pathogens

The application of histological methods for the detection of pathogens in ancient tissues has provided information for better understanding the origin and spread of infectious diseases [6]. Currently, the representation of past epidemics relies on molecular biology, which may be an approach limited to pathogens in the event that the altered DNA prevents the amplification of pathogen-specific DNA sequences. Immunohistochemistry has been successfully used in a number of paleopathological studies, including the detection of bacteria such as *T. whipplei* and *R. rickettsii* in paraffin blocks at least a century old from autopsy cases [22, 23], the sporadic typhus agent *Rickettsia typhi* in paraffin-embedded tissue blocks from the Second World War (Hamburg, Germany, 1940-1944) [228], and the detection of a parasite (*Taenia solium*) from stomach tissue sections dating from the Ptolemaic period [21]. These studies demonstrated that the antigenicity of proteins could be preserved in ancient tissues. We invented microbial paleoserology, to reveal an epidemic of recurrent fever in a 16^th^-17^th^ century French garrison, which had been missed by real-time PCR [229]. This demonstrates the interest of applying other complementary approaches allowing the detection of antigens or antibodies specific to the pathogens. In that pioneering work, total immunoglobulins previously demonstrated in ancient dental pulp [230], were extracted and applied to inactivated pathogens used as antigens in a so-called “mini blot” format [229]. Extending these observations to the detection of unidentified pathogens, we are now applying auto-immunohistochemistry used for the detection of pathogens in modern-day tissues, to ancient samples, mainly dental pulp. We call this new, antigen-based detection “paleo-autoimmunohistochemistry”.

## Supplementary Information

**Additional file 1: Supplementary Material**

## Data Availability

The data that support of this study are available from the corresponding author upon reasonable request.

## Abbreviations

IHC: Immunohistochemistry
PAP: Peroxidase-antiperoxidase technique
ABC: Avidin-biotin peroxidase complex
LAB: Peroxidase-labelled avidin-biotin
TGF-α: Transforming growth factor alpha
*B. quintana*: *Bartonella quintana*
*Y. pestis*: *Yersinia pestis*
*T. pallidum*: *Treponema pallidum*
*C. trachomatis*: *Chlamydia trachomatis*
HHV: Human Herpesviruses
VZV: Varicella-zoster virus
HSV: Herpes simplex virus
EBV: Epstein-Barr virus
KS: Kaposi’s sarcoma
CMV: Cytomegalovirus
HIV: Human Immunodeficiency Virus
AIDS: Acquired Immunodeficiency Syndrome
MERS-CoV: Middle East respiratory syndrome coronavirus
SARS: Severe acute respiratory syndrome

## References

[1] Fournier P-E, Drancourt M, Colson P, Rolain J-M, La Scola B, Raoult D (2013). Modern clinical microbiology: new challenges and solutions. Nat Rev Microbiol..

[2] Eyzaguirre E, Haque AK (2008). Application of Immunohistochemistry to Infections. Arch Pathol Lab Med..

[3] Fellag M, Saad J, Armstrong N, Chabrière E, Eldin C, Lagier J-C (2019). Routine Culture-Resistant Mycobacterium tuberculosis Rescue and Shell-Vial Assay. Fr Emerg Infect Dis..

[4] Hawes D, Shi S-R, Dabbs DJ, Taylor CR, Cote RJ, Weidner N, Cote RJ, Suster S, Weiss LM (2009). CHAPTER 5 - Immunohistochemistry. Modern Surgical Pathology.

[5] Molina-Ruiz AM, Cerroni L, Kutzner H, Requena L (2015). Immunohistochemistry in the Diagnosis of Cutaneous Bacterial Infections. Am J Dermatopathol.

[6] Raoult D, Drancourt M (2008). éditeurs. Paleomicrobiology: past human infections.

[7] Roskams T (2002). The role of immunohistochemistry in diagnosis. Clin Liver Dis..

[8] Shi SR, Key ME, Kalra KL (1991). Antigen retrieval in formalin-fixed, paraffin-embedded tissues: an enhancement method for immunohistochemical staining based on microwave oven heating of tissue sections. J Histochem Cytochem..

[9] Werner M, Chott A, Fabiano A, Battifora H (2000). Effect of Formalin Tissue Fixation and Processing on Immunohistochemistry. Am J Surg Pathol.

[10] Grillo F, Bruzzone M, Pigozzi S, Prosapio S, Migliora P, Fiocca R (2017). Immunohistochemistry on old archival paraffin blocks: is there an expiry date?. J Clin Pathol..

[11] Coons AH, Creech HJ, Jones RN (1941). Immunological Properties of an Antibody Containing a Fluorescent Group. Exp Biol Med..

[12] Nakane PK, Pierce GB (1966). Enzyme-labeled antibodies: Preparation and application for the localization of antigens. J Histochem Cytochem..

[13] Sternberger LA, Hardy PH, Cuculis JJ, Meyer HG (1970). The unlabeled antibody enzyme method of immunohistochemistry preparation and properties of soluble antigen-antibody complex (Horseradish peroxidase-antihorseradish peroxidase) and its use in identification of spirochetes. J Histochem Cytochem..

[14] Mason DY, Sammons R (1978). Alkaline phosphatase and peroxidase for double immunoenzymatic labelling of cellular constituents. J Clin Pathol..

[15] Guesdon JL, Ternynck T, Avrameas S (1979). The use of avidin-biotin interaction in immunoenzymatic techniques. J Histochem Cytochem..

[16] Hsu SM, Raine L, Fanger H (1981). Utilisation du complexe avidine-biotine-peroxydase (ABC) dans les techniques d’immunoperoxydase: une comparaison entre les procédures ABC et les anticorps non marqués (PAP). J Histochem Cytochem..

[17] Elias JM, Margiotta M, Gaborc D (1989). Sensitivity and Detection Efficiency of the Peroxidase Antiperoxidase (PAP), Avidin–Biotin Peroxidase Complex (ABC), and Peroxidase-Labeled Avidin–Biotin (LAB) Methods. Am J Clin Pathol..

[18] Sn H (1976). H M, Jd M. Application of immunofluorescent staining on paraffin sections improved by trypsin digestion. Lab Invest..

[19] Brigati DJ, Budgeon LR, Unger ER, Koebler D, Cuomo C, Kennedy T (1988). Immunocytochemistry is Automated: Development of A Robotic Workstation Based Upon the Capillary Action Principle. J Histotechnol..

[20] Shi SR, Key ME, Kalra KL (1991). Récupération d’antigène dans les tissus fixés au formol, inclus en paraffine: une méthode d’amélioration de la coloration immunohistochimique basée sur le chauffage au four à micro-ondes de coupes de tissus. J Histochem Cytochem..

[21] Bruschi F, Masetti M, Locci MT, Ciranni R, Fornaciari G (2006). Short report: cysticercosis in an Egyptian mummy of the late Ptolemaic period. Am J Trop Med Hyg..

[22] Dumler JS (1991). Fatal Rocky Mountain Spotted Fever in Maryland—1901. JAMA..

[23] Dumler JS, Baisden BL, Yardley JH, Raoult D (2003). Immunodetection of *Tropheryma whipplei* in Intestinal Tissues from Dr. Whipple’s 1907 Patient. N Engl J Med..

[24] Herman GE, Elfont EA, Floyd AD, Javois LC, Overview of Automated Immunostainers (1995). Immunocytochemical Methods and Protocols.

[25] Le Neel T, Moreau A, Laboisse C, Truchaud A (1998). Comparative evaluation of automated systems in immunohistochemistry. Clinica Chimica Acta..

[26] Moreau A, Le Neel T, Joubert M, Truchaud A, Laboisse C (1998). Approach to automation in immunohistochemistry. Clinica Chimica Acta..

[27] Linnoila I, Petrusz P (1984). Immunohistochemical techniques and their applications in the histopathology of the respiratory system. Environ Health Perspect..

[28] Bratthauer GL, Oliver C, Jamur MC, The Avidin–Biotin Complex (ABC) Method and Other Avidin–Biotin Binding Methods (2010). Immunocytochemical Methods and Protocols.

[29] Bussolati G, Leonardo E (2008). Technical pitfalls potentially affecting diagnoses in immunohistochemistry. J Clin Pathol..

[30] Dapson RW (1993). Fixation for the 1990’s: a Review of Needs and Accomplishments. Biotechnic Histochem..

[31] Ramos-Vara JA, Miller MA (2014). When Tissue Antigens and Antibodies Get Along: Revisiting the Technical Aspects of Immunohistochemistry—The Red, Brown, and Blue Technique. Vet Pathol..

[32] Goldstein NS, Ferkowicz M, Odish E, Mani A, Hastah F (2003). Minimum Formalin Fixation Time for Consistent Estrogen Receptor Immunohistochemical Staining of Invasive Breast Carcinoma. Am J Clin Pathol..

[33] Leong AS-Y, Gilham PN (1989). The effects of progressive formaldehyde fixation on the preservation of tissue antigens. Pathology..

[34] Montero C (2003). The Antigen-Antibody Reaction in Immunohistochemistry. J Histochem Cytochem..

[35] Eltoum I, Fredenburgh J, Myers RB, Grizzle WE (2001). Introduction to the Theory and Practice of Fixation of Tissues. J Histotechnol..

[36] Arnold MM, Srivastava S, Fredenburgh J, Stockard CR, Myers RB, Grizzle WE (1996). Effects of Fixation and Tissue Processing on Immunohistochemical Demonstration of Specific Antigens. Biotechnic Histochem..

[37] Grizzle WE, Stockard CR, Billings PE (2001). The Effects of Tissue Processing Variables Other Than Fixation on Histochemical Staining and Immunohistochemical Detection of Antigens. J Histotechnol..

[38] Leong AS-Y, Milios J, Duncis CG (1988). Antigen preservation in microwave-irradiated tissues: A comparison with formaldehyde fixation. J Pathol..

[39] Prichard JW (2014). Overview of Automated Immunohistochemistry. Arch Pathol Lab Med..

[40] Maurin M, Raoult D (1996). *Bartonella* (*Rochalimaea*) *quintana* infections. Clin Microbiol Rev..

[41] Li JS, Sexton DJ, Mick N, Nettles R, Fowler VG, Ryan T (2000). Proposed Modifications to the Duke Criteria for the Diagnosis of Infective Endocarditis. Clin Infect Dis..

[42] Lepidi H, Fournier P-E, Raoult D (2000). Quantitative Analysis of Valvular Lesions During Bartonella Endocarditis. Am J Clin Pathol.

[43] Lepidi H, Durack DT, Raoult D (2002). Diagnostic methods current best practices and guidelines for histologic evaluation in infective endocarditis. Infect Dis Clin North Am..

[44] Barbieri R, Signoli M, Chevé D, Costedoat C, Tzortzis S, Aboudharam G (2020). *Yersinia pestis* : the Natural History of Plague. Clin Microbiol Rev.

[45] Engelthaler DM, Gage KL, Montenieri JA, Chu M, Carter LG (1999). PCR Detection of Yersinia pestis in Fleas: Comparison with Mouse Inoculation. J Clin Microbiol..

[46] Ratsitorahina M, Chanteau S, Rahalison L, Ratsifasoamanana L, Boisier P (2000). Epidemiological and diagnostic aspects of the outbreak of pneumonic plague in Madagascar. Lancet..

[47] Guarner J, Shieh W-J, Greer PW, Gabastou J-M, Chu M, Hayes E (2002). Immunohistochemical Detection of *Yersinia pestis* in Formalin-Fixed. Paraffin-Embedded Tissue. Am J Clin Pathol..

[48] Gabastou J-M, Proaño J, Vimos A, Jaramillo G, Hayes E, Gage K (2000). An outbreak of plague including cases with probable pneumonic infection, Ecuador, 1998. Trans R Soc Trop Med Hyg..

[49] Majander K, Pfrengle S, Kocher A, Neukamm J, L du P, Pla-Díaz M (2020). Ancient Bacterial Genomes Reveal a High Diversity of *Treponema pallidum* Strains in Early Modern Europe. Curr Biol.

[50] Belkacemi S, Khalil JB, Ominami Y, Hisada A, Fontanini A, Caputo A, et al. Passive Filtration, Rapid Scanning Electron Microscopy, and Matrix-Assisted Laser Desorption Ionization–Time of Flight Mass Spectrometry for Treponema Culture and Identification from the Oral Cavity. J Clin Microbiol. 2019;57(10) https://jcm.asm.org/content/57/10/e00517-19. 10.1128/JCM.00517-19 PMC6760945 31340994

[51] Edmondson DG, Hu B, Norris SJ. Long-Term In Vitro Culture of the Syphilis Spirochete *Treponema pallidum* subsp. pallidum. mBio. 2018;9(3) https://mbio.asm.org/content/9/3/e01153-18. 10.1128/mBio.01153-18 PMC6020297 29946052

[52] Hoang MP, High WA, Molberg KH (2004). Secondary syphilis: a histologic and immunohistochemical evaluation. J Cutan Pathol..

[53] Martín-Ezquerra G, Fernandez-Casado A, Barco D, Jucglà A, Juanpere-Rodero N, Manresa JM (2009). *Treponema pallidum* distribution patterns in mucocutaneous lesions of primary and secondary syphilis: an immunohistochemical and ultrastructural study. Hum Pathol..

[54] Phelps RG, Knispel J, Tu ES, Cernainu G, Saruk M (2000). Immunoperoxidase technique for detecting spirochetes in tissue sections: comparison with other methods. Int J Dermatol..

[55] Tse JY, Chan MP, Ferry JA, Deshpande V, Sohani AR, Nardi V (2018). Syphilis of the Aerodigestive Tract. Am J Surg Pathol.

[56] Fernandez-Flores A (2010). Immunostaining for *Treponema pallidum*: Caution in its Evaluation. Am J Dermatopathol..

[57] Siqueira CS, Saturno JL, de Sousa SCOM, da Silveira FRX (2014). Diagnostic approaches in unsuspected oral lesions of syphilis. Int J Oral Maxillofac Surg..

[58] Quatresooz P, Piérard GE (2009). Skin Homing of *Treponema pallidum* in Early Syphilis: An Immunohistochemical Study. Appl Immunohistochem Mol Morphol..

[59] Calvo DF, Cassarino D, Fernandez-Flores A (2020). Syphilitic Chancre of the Lip. Am J Dermatopathol..

[60] Kenny B, Hamza S, Peermohamed S, Shumilak G, Groot G, Osmond A (2021). A case of a pseudoneoplastic primary syphilis chancre on the neck. JAAD Case Rep..

[61] Veasey JV, Lellis RF, MFF de C B, Porto PL, JCS C (2016). Papulonodular secondary syphilis: a rare clinic presentation confirmed by serologic and histologic exams. An Bras Dermatol..

[62] Cid PM, Cudós ES, Zamora Vargas FX, Merino MJB, Pinto PH (2014). Pathologically Confirmed Malignant Syphilis Using Immunohistochemical Staining: Report of 3 Cases and Review of the Literature. Sex Transm Dis..

[63] Chiang M-C, Chiang F-C, Chang Y-T, Chen T-L, Fung C-P (2010). Erythema Multiforme Caused by *Treponema pallidum* in a Young Patient with Human Immunodeficiency Virus Infection. J Clin Microbiol..

[64] Buffet M, Grange PA, Gerhardt P, Carlotti A, Calvez V, Bianchi A (2007). Diagnosing Treponema pallidum in Secondary Syphilis by PCR and Immunohistochemistry. J Invest Dermatol..

[65] Chu C-Y, Chen W-Y, Yeh S-D, Yeh H-M, Fang C-L. Syphilitic orchitis mimicking a testicular tumor in a clinically occult HIV-infected young man: a case report with emphasis on a challenging pathological diagnosis. Diagn Pathol. 2016;11 https://www.ncbi.nlm.nih.gov/pmc/articles/PMC4712524/. 10.1186/s13000-016-0454-x PMC4712524 26762155

[66] Debonnet C, Robin G, Prasivoravong J, Vuotto F, Catteau-Jonard S, Faure K, et al. Infection à *Chlamydia trachomatis* : mise au point. Gynécologie Obstétrique Fertilité & Sénologie. 2021;S2468718921000040. 10.1016/j.gofs.2021.01.003 33434747

[67] Unemo M, Bradshaw CS, Hocking JS, de Vries HJC, Francis SC, Mabey D (2017). Sexually transmitted infections: challenges ahead. Lancet Infect Dis..

[68] Feltes F, Vallés L, Alcaraz I, Kutzner H, Requena L. Lymphogranuloma Venereum: Report of Two Cases with « Bubonulus » As Primary Stage and Immunohistochemical Demonstration of Chlamydia Trachomatis. 2015;6(1):3.

[69] Bryan ER, McLachlan RI, Rombauts L, Katz DJ, Yazdani A, Bogoevski K (2019). Detection of chlamydia infection within human testicular biopsies. Hum Reprod..

[70] Noguchi Y, Yabushita H, Noguchi M, Fujita M, Asai M, Del Carpio CA (2002). Detection of Chlamydia trachomatis infection with DNA extracted from formalin-fixed paraffin-embedded tissues. Diagn Microbiol Infect Dis..

[71] Lepidi H, Coulibaly B, Casalta J-P, Raoult D (2006). Autoimmunohistochemistry: A New Method for the Histologic Diagnosis of Infective Endocarditis. J Infect Dis..

[72] Lepidi H, Fenollar F, Dumler JS, Gauduchon V, Chalabreysse L, Bammert A (2004). Cardiac Valves in Patients with Whipple Endocarditis: Microbiological, Molecular, Quantitative Histologic, and Immunohistochemical Studies of 5 Patients. J Infect Dis..

[73] Lepidi H, Houpikian P, Liang Z, Raoult D (2003). Cardiac Valves in Patients with Q Fever Endocarditis: Microbiological, Molecular, and Histologic Studies. J Infect Dis..

[74] Brouqui P, Raoult D (2001). Endocarditis Due to Rare and Fastidious Bacteria. Clin Microbiol Rev..

[75] Lamas CC, Eykyn SJ (2003). Blood culture negative endocarditis: analysis of 63 cases presenting over 25 years. Heart..

[76] Fournier PE, Raoult D (1999). Nonculture Laboratory Methods for the Diagnosis of Infectious Endocarditis. Curr Infect Dis Rep..

[77] Baisden BL, Lepidi H, Raoult D, Argani P, Yardley JH, Dumler JS (2002). Diagnosis of Whipple Disease by Immunohistochemical Analysis A Sensitive and Specific Method for the Detection of *Tropheryma whipplei* (the Whipple Bacillus) in Paraffin-Embedded Tissue. Am J Clin Pathol..

[78] Fournier P-E, Thuny F, Richet H, Lepidi H, Casalta J-P, Arzouni J-P (2010). Comprehensive Diagnostic Strategy for Blood Culture-Negative Endocarditis: A Prospective Study of 819 New Cases. Clin Infect Dis..

[79] Guarner J, Bartlett J, Reagan S, Fischer M, Finn S, Obriain D (2006). Immunohistochemical evidence of Clostridium sp, *Staphylococcus aureus*, and group A Streptococcus in severe soft tissue infections related to injection drug use. Hum Pathol..

[80] Baker C, Sriharan A (2020). Pattern of Cross-Reactivity Between Mycobacterial Immunohistochemical Stain and Normal Human Eosinophils: A Potential Pitfall in the Diagnosis of Cutaneous Mycobacterial Infections. Am J Dermatopathol..

[81] Goel MM, Budhwar P (2007). Immunohistochemical localization of *Mycobacterium tuberculosis complex* antigen with antibody to 38 kDa antigen versus Ziehl Neelsen staining in tissue granulomas of extrapulmonary tuberculosis. Indian J Tuberc..

[82] Kohli R, Punia RS, Kaushik R, Kundu R, Mohan H (2014). Relative value of immunohistochemistry in detection of mycobacterial antigen in suspected cases of tuberculosis in tissue sections. Indian J Pathol Microbiol..

[83] Solomon IH, Johncilla ME, Hornick JL, Milner DA (2017). The Utility of Immunohistochemistry in Mycobacterial Infection: A Proposal for Multimodality Testing. Am J Surg Pathol..

[84] Verhagen C, Faber W, Klatser P, Buffing A, Naafs B, Das P (1999). Immunohistological Analysis of In Situ Expression of Mycobacterial Antigens in Skin Lesions of Leprosy Patients Across the Histopathological Spectrum. Am J Pathol..

[85] Talhari S, de Souza Santos MN, Talhari C, de Lima Ferreira LC, Silva RM, Zelger B (2010). *Borrelia Burgdorferi* “sensu lato” in Brazil: Occurrence confirmed by immunohistochemistry and focus floating microscopy. Acta Tropica..

[86] Lash RH, Genta RM (2016). Routine Anti- Helicobacter Immunohistochemical Staining is Significantly Superior to Reflex Staining Protocols for the Detection of *Helicobacter* in Gastric Biopsy Specimens. Helicobacter..

[87] Shukla S, Pujani M, Agarwal A, Pujani M, Rohtagi A (2012). Correlation of Serology with Morphological Changes in Gastric Biopsy in *Helicobacter pylori* Infection and Evaluation of Immunohistochemistry for *H. pylori* Identification. Saudi J Gastroenterol..

[88] Soylu A, Ozkara S, Alıs H, Dolay K, Kalaycı M, Yasar N (2008). Immunohistochemical testing for *Helicobacter Pylori* existence in neoplasms of the colon. BMC Gastroenterol..

[89] Toulaymat M, Marconi S, Garb J, Otis C, Nash S (1999). Endoscopic Biopsy Pathology of *Helicobacter pylori* Gastritis: Comparison of Bacterial Detection by Immunohistochemistry and Genta Stain. Arch Pathol Lab Med..

[90] Ngaiza AI, Yahaya JJ, Mwakimonga AK, Vuhahula E, Mnango L, Mwakigonja AR (2021). Histologic detection of *Helicobacter pylori* by the immunohistochemical method using anti-*Helicobacter pylori* polyclonal antibody: A cross-sectional study of patients with gastric pathologies at the Muhimbili National Hospital in Dar-es-salaam.

[91] Paddock CD, Greer PW, Ferebee TL, Singleton J, McKechnie DB, Treadwell TA (1999). Hidden Mortality Attributable to Rocky Mountain Spotted Fever: Immunohistochemical Detection of Fatal, Serologically Unconfirmed Disease. J Infect Dis..

[92] Quintero Vélez JC, Faccini-Martínez ÁA, Rodas González JD, Díaz FJ, Ramírez García R, Somoyar Ordosgoitia P (2019). Fatal *Rickettsia rickettsii* infection in a child, Northwestern Colombia, 2017. Ticks Tick-borne Dis..

[93] Zavala-Castro JE, Zavala-Velázquez JE, Walker DH, Arcila EER, Laviada-Molina H, Olano JP (2006). Fatal Human Infection with *Rickettsia rickettsii,* Yucatán. Mexico. Emerg Infect Dis..

[94] Juvonen J, Laurila A, Juvonen T, Alakärppä H, Surcel H-M, Lounatmaa K (1997). Detection of *Chlamydia pneumoniae* in Human Nonrheumatic Stenotic Aortic Valves. J Am Coll Cardiol..

[95] Wohlschlaeger J, Wimmer MLJ, Nägler DK, Haberl R, Weis S (2005). Identification of *Chlamydia pneumoniae* in intracranial and extracranial arteries in patients with stroke and in controls: combined immunohistochemical and polymerase chain reaction analyses☆. Hum Pathol..

[96] Chung J-H, Lim S-C, Yun N-R, Shin S-H, Kim C-M, Kim D-M (2012). Scrub typhus hepatitis confirmed by immunohistochemical staining. World J Gastroenterol..

[97] Kim D-M, Park C-J, Lim S-C, Park K-H, Jang W-J, Lee S-H (2008). Diagnosis of Scrub Typhus by Immunohistochemical Staining of *Orientia tsutsugamushi* in Cutaneous Lesions. Am J Clin Pathol..

[98] Kim D-M, Lim S-C, Won KJ, Choi Y-J, Park K-H, Jang W-J (2007). Severe Scrub Typhus Confirmed Early via Immunohistochemical Staining. Am J Trop Med Hyg..

[99] Tseng B-Y, Yang H-H, Liou J-H, Chen L-K, Hsu Y-H (2008). Immunohistochemical Study of Scrub Typhus: A Report of Two Cases. Kaohsiung J Med Sci..

[100] Guarner J, Greer PW, Whitney A, Shieh W-J, Fischer M, White EH (2004). Pathogenesis and Diagnosis of Human Meningococcal Disease Using Immunohistochemical and PCR Assays. Am J Clin Pathol..

[101] Ogata S, Shimizu K, Oda T, Tominaga S, Nakanishi K (2016). Immunohistochemical detection of human intestinal spirochetosis. Hum Pathol..

[102] Wong KT, Vadivelu J, Puthucheary SD, Tan KL (1996). An immunohistochemical method for the diagnosis of melioidosis. Pathology..

[103] Zuckerman AJ (2004). éditeur. Principles and practice of clinical virology. 5th ed.

[104] Bharucha T, Houlihan CF, Breuer J (2019). Herpesvirus Infections of the Central Nervous System. Semin Neurol..

[105] Chayavichitsilp P, Buckwalter JV, Krakowski AC, Friedlander SF (2009). Herpes Simplex. Pediatr Rev..

[106] Nikkels AF, Delvenne P, Sadzot-Delvaux C, Debrus S, Piette J, Rentier B (1996). Distribution of varicella zoster virus and herpes simplex virus in disseminated fatal infections. J Clin Pathol..

[107] Strickler JG, Manivel JC, Copenhaver CM, Kubic VL (1990). Comparison of in situ hybridization and immunohistochemistry for detection of cytomegalovirus and herpes simplex virus. Hum Pathol..

[108] Nikkels AF, Debrus S, Sadzot-Delvaux C, Piette J, Delvenne P, Rentier B (1993). Comparative immunohistochemical study of herpes simplex and varicella-zoster infections. Vichows Archiv A Pathol Anat..

[109] Mescher T, Boyer PJ, Bubak AN, Hassell JE, Nagel MA (2021). Detection of varicella zoster virus antigen and DNA in two cases of cerebral amyloid angiopathy. J Neurol Sci..

[110] Jazeron J-F, Barbe C, Frobert E, Renois F, Talmud D, Brixi-Benmansour H (2012). Virological Diagnosis of Herpes Simplex Virus 1 Esophagitis by Quantitative Real-Time PCR Assay. J Clin Microbiol..

[111] Kurokawa I, Yamamoto M, Kurata T. Varicella Zoster Virus Antigens in the Epidermis of Patients with Herpes Zoster before and after Treatment with Acyclovir: An Immunohistochemical Study. J Int Med Res. 2016; https://journals.sagepub.com/doi/10.1177/147323000102900307?url_ver=Z39.88-2003&rfr_id=ori%3Arid%3Acrossref.org&rfr_dat=cr_pub++0pubmed. 10.1177/147323000102900307 11471857

[112] Leinweber B, Kerl H, Cerroni L (2006). Histopathologic Features of Cutaneous Herpes Virus Infections (Herpes Simplex, Herpes Varicella/Zoster): A Broad Spectrum of Presentations With Common Pseudolymphomatous Aspects. Am J Surg Pathol..

[113] Nikkels AF, Debrus S, Sadzot-Delvaux C, Piette J, Rentier B, Piérard GE (1995). Immunohistochemical identification of varicella-zoster virus gene 63-encoded protein (IE63) and late (gE) protein on smears and cutaneous biopsies: Implications for diagnostic use. J Med Virol..

[114] Nazzaro G, Veraldi S (2020). Herpes zoster incognito: an immunohistochemical diagnosis. Anais Brasileiros de Dermatologia..

[115] Makino K, Takeichi O, Hatori K, Imai K, Ochiai K, Ogiso B (2015). Epstein-Barr virus infection in chronically inflamed periapical granulomas. PLoS ONE..

[116] Saboia-Dantas CJ (2007). Coutrin de Toledo LF, Sampaio-Filho HR, Siqueira JF. Herpesviruses in asymptomatic apical periodontitis lesions: an immunohistochemical approach. Oral Microbiol Immunol..

[117] Baizig NM, Wided BA, Amine OE, Gritli S, ElMay M (2020). The Clinical Significance of IGF-1R and Relationship with Epstein–Barr Virus Markers: LMP1 and EBERs in Tunisian Patients with Nasopharyngeal Carcinoma. Ann Otol Rhinol Laryngol..

[118] Maiese A, La Russa R, Passaro G, Santoro P, De Matteis A, Fineschi V (2020). Fatal Epstein-Barr virus infection in an immunocompetent host: a postmortem diagnosis. Forensic Sci Med Pathol..

[119] Ishtiaq S, Hassan U, Mushtaq S, Akhtar N (2013). Determination of Frequency of Epstein-Barr Virus in Non-Hodgkin Lymphomas Using EBV Latent Membrane Protein 1 (EBV-LMP1) Immunohistochemical Staining. Asian Pac J Cancer Prev..

[120] Massini G, Siemer D, Hohaus S. EBV in Hodgkin Lymphoma. Mediterr J Hematol Infect Dis. 2009;1(2) https://www.ncbi.nlm.nih.gov/pmc/articles/PMC3033177/. 10.4084/MJHID.2009.013 PMC3033177 21416003

[121] Valente G, Secchiero P, Lusso P, Abete MC, Jemma C, Reato G (1996). Human herpesvirus 6 and Epstein-Barr virus in Hodgkin’s disease: a controlled study by polymerase chain reaction and in situ hybridization. Am J Pathol..

[122] Chang Y, Cesarman E, Pessin M, Lee F, Culpepper J, Knowles D (1994). Identification of herpesvirus-like DNA sequences in AIDS-associated Kaposi’s sarcoma. Science..

[123] Cheuk W, Wong KOY, Wong CSC, Dinkel JE, Ben-Dor D, Chan JKC (2004). Immunostaining for Human Herpesvirus 8 Latent Nuclear Antigen-1 Helps Distinguish Kaposi Sarcoma From Its Mimickers. Am J Clin Pathol..

[124] Chadburn A, Wilson J, Wang YL. Molecular and Immunohistochemical Detection of Kaposi Sarcoma Herpesvirus/Human Herpesvirus-8. In: Czader M, éditeur. Hematological Malignancies. Totowa, NJ: Humana Press; 2013. 245-256. (Methods in Molecular Biology; vol. 999). http://link.springer.com/10.1007/978-1-62703-357-2_18 10.1007/978-1-62703-357-2_18 23666704

[125] Hong A, Davies S, Soon LC (2003). Immunohistochemical detection of the human herpes virus 8 (HHV8) latent nuclear antigen-1 in Kaposi’s sarcoma. Pathology..

[126] Patel RM, Goldblum JR, Hsi ED (2004). Immunohistochemical detection of human herpes virus-8 latent nuclear antigen-1 is useful in the diagnosis of Kaposi sarcoma. Mod Pathol..

[127] Pereira PF, Cuzzi T, Galhardo MCG (2013). Immunohistochemical detection of the latent nuclear antigen-1 of the human herpesvirus type 8 to differentiate cutaneous epidemic Kaposi sarcoma and its histological simulators*. An Bras Dermatol..

[128] Robin Y-M, Guillou L, Michels J-J, Coindre J-M (2004). Human Herpesvirus 8 Immunostaining: A Sensitive and Specific Method for Diagnosing Kaposi Sarcoma in Paraffin-Embedded Sections. Am J Clin Pathol..

[129] Schwartz EJ, Dorfman RF, Kohler S (2003). Human Herpesvirus-8 Latent Nuclear Antigen-1 Expression in Endemic Kaposi Sarcoma: An Immunohistochemical Study of 16 Cases. Am J Surg Pathol..

[130] Anwar F, Erice A, Jessurun J (1999). Are there cytopathic features associated with cytomegalovirus infection predictive of resistance to antiviral therapy?. Annals of Diagnostic Pathology..

[131] Juric-Sekhar G, Upton MP, Swanson PE, Westerhoff M (2017). Cytomegalovirus (CMV) in gastrointestinal mucosal biopsies: should a pathologist perform CMV immunohistochemistry if the clinician requests it?. Hum Pathol..

[132] Mills AM, Guo FP, Copland AP, Pai RK, Pinsky BA (2013). A Comparison of CMV Detection in Gastrointestinal Mucosal Biopsies Using Immunohistochemistry and PCR Performed on Formalin-fixed, Paraffin-embedded Tissue. Am J Surg Pathol..

[133] Rimsza LM, Vela EE, Frutiger YM, Rangel CS, Solano M, Richter LC (1996). Rapid Automated Combined In Situ Hybridization and Immunohistochemistry for Sensitive Detection of Cytomegalovirus in Paraffin-Embedded Tissue Biopsies. Am J Clin Pathol..

[134] Sheehan MM, Coker R, Coleman DV (1998). Detection of cytomegalovirus (CMV) in HIV+ patients: comparison of cytomorphology, immunocytochemistry and in situ hybridization. Cytopathology..

[135] Lu DY, Qian J, Easley KA, Waldrop SM, Cohen C (2009). Automated In Situ Hybridization and Immunohistochemistry for Cytomegalovirus Detection in Paraffin-embedded Tissue Sections. Appl Immunohistochem Mol Morphol.

[136] Kutza AST, Muhl E, Hackstein H, Kirchner H, Bein G (1998). High Incidence of Active Cytomegalovirus Infection Among Septic Patients. CLIN INFECT DIS..

[137] Iwasenko JM, Howard J, Arbuckle S, Graf N, Hall B, Craig ME (2011). Human Cytomegalovirus Infection Is Detected Frequently in Stillbirths and Is Associated With Fetal Thrombotic Vasculopathy. J Infect Dis..

[138] Saetta A, Agapitos E, Davaris PS (1998). Determination of CMV placentitis. Virchows Archiv..

[139] Ribalta T, Martinez JA, Jares P, Muntané J, Miquel R, Claramonte X (2002). Presence of occult cytomegalovirus infection in the brain after orthotopic liver transplantation: An autopsy study of 83 cases. Virchows Arch..

[140] De Marchi Andrade ZR, Garippo AL, Saldiva PHN, Capelozzi VL (2004). Immunohistochemical and in situ detection of cytomegalovirus in lung autopsies of children immunocompromised by secondary interstitial pneumonia. Pathol Res Pract..

[141] Genta RM, Bleyzer I, Cate TR, Tandon AK, Yoffe B (1993). In situ hybridization and immunohistochemical analysis of cytomegalovirus-associated ileal perforation. Gastroenterology..

[142] Hazır-Konya H, Avkan-Oğuz V, Akpınar H, Sağol Ö, Sayıner A (2021). Investigation of Cytomegalovirus in Intestinal Tissue in a Country With High CMV Seroprevalence. Turk J Gastroenterol..

[143] Solomon IH, Hornick JL, Laga AC (2017). Immunohistochemistry Is Rarely Justified for the Diagnosis of Viral Infections. Am J Clin Pathol..

[144] Mitchell DA, Xie W, Schmittling R, Learn C, Friedman A, McLendon RE (2008). Sensitive detection of human cytomegalovirus in tumors and peripheral blood of patients diagnosed with glioblastoma. Neuro Oncol..

[145] Kambham N, Vij R, Cartwright CA, Longacre T (2004). Cytomegalovirus Infection in Steroid-refractory Ulcerative Colitis. Am J Surg Pathol..

[146] Hasegawa T, Aomatsu K, Nakamura M, Aomatsu N, Aomatsu K (2015). Cytomegalovirus colitis followed by ischemic colitis in a non-immunocompromised adult: A case report. World J Gastroenterol..

[147] Salahuddin SZ, Ablashi DV, Markham PD, Josephs SF, Sturzenegger S, Kaplan M (1986). Isolation of a new virus, HBLV, in patients with lymphoproliferative disorders. Science..

[148] Bai Y, Wang Z, Sun K, Yao H (2014). HHV-6-associated acute lymphadenitis in immunocompetent patients: a case report and review of literature. Int J Clin Exp Pathol..

[149] Maric I, Bryant R, Abu-Asab M, Cohen JI, Vivero A, Jaffe ES (2004). Human herpesvirus-6-associated acute lymphadenitis in immunocompetent adults. Mod Pathol..

[150] Hoshino K, Nishi T, Adachi H, Ito H, Fukuda Y, Dohi K (1995). Human herpesvirus-6 infection in renal allografts: retrospective immunohistochemical study in Japanese recipients. Transplant Int..

[151] Carrigan DR, Tapper MA, Knox KK, Drobyski WR, Ash RC, Russler SK (1991). Interstitial pneumonitis associated with human herpesvirus-6 infection after marrow transplantation. Lancet..

[152] Knox KK, Brewer JH, Henry JM, Harrington DJ, Carrigan DR (2000). Human Herpesvirus 6 and Multiple Sclerosis: Systemic Active Infections in Patients with Early Disease. Clin Infect Dis..

[153] Skuja S, Svirskis S, Murovska M (2021). Human Herpesvirus-6 and -7 in the Brain Microenvironment of Persons with Neurological Pathology and Healthy People. Int J Mol Sci..

[154] Knox KK, Harrington DP, Carrigan DR (1995). Fulminant human herpesvirus six encephalitis in a human immunodeficiency virus-infected infant. J Med Virol..

[155] Razzaque A, Francillon Y, Jilly PN, Varricchio F (1996). Detection of human herpesvirus 6 sequences in lymphoma tissues by immunohistochemistry and polymerase chain reactions. Cancer Lett..

[156] Blauvelt A (2001). Skin Diseases Associated with Human Herpesvirus 6, 7, and 8 Infection. J Invest Dermatol Symp Proc..

[157] Pantry SN, Medveczky PG. Latency, Integration, and Reactivation of Human Herpesvirus-6. Viruses. 2017;9(7) https://www.ncbi.nlm.nih.gov/pmc/articles/PMC5537686/. 10.3390/v9070194 PMC5537686 28737715

[158] Frenkel N, Schirmer EC, Wyatt LS, Katsafanas G, Roffman E, Danovich RM (1990). Isolation of a new herpesvirus from human CD4+ T cells. PNAS..

[159] Kempf W, Adams V, Mirandola P, Menotti L, Di Luca D, Wey N (1998). Persistence of Human Herpesvirus 7 in Normal Tissues Detected by Expression of a Structural Antigen. J Infect Dis..

[160] Ward JM, O’Leary TJ, Baskin GB, Benveniste R, Harris CA, Nara PL (1987). Immunohistochemical localization of human and simian immunodeficiency viral antigens in fixed tissue sections. Am J Pathol..

[161] de Paiva GR, Laurent C, Godel A, da Silva NA, March M, Delsol G (2007). Discovery of Human Immunodeficiency Virus Infection by Immunohistochemistry on Lymph Node Biopsies From Patients With Unexplained Follicular Hyperplasia. Am J Surg Pathol.

[162] Jarry A, Cortez A, René E, Muzeau F, Brousse N (2007). Infected cells and immune cells in the gastrointestinal tract of AIDS patients. An immunohistochemical study of 127 cases. Histopathology..

[163] Pomerantz RJ (1988). Human Immunodeficiency Virus (HIV) Infection of the Uterine Cervix. Ann Intern Med..

[164] Sekikawa Y, Hongo I. HIV-associated benign lymphoepithelial cysts of the parotid glands confirmed by HIV-1 p24 antigen immunostaining. BMJ Case Rep. 2017; https://www.ncbi.nlm.nih.gov/pmc/articles/PMC5652621/. 10.1136/bcr-2017-221869 PMC5652621 28963391

[165] Moonim MT, Alarcon L, Freeman J, Mahadeva U, van der Walt JD, Lucas SB (2010). Identifying HIV infection in diagnostic histopathology tissue samples - the role of HIV-1 p24 immunohistochemistry in identifying clinically unsuspected HIV infection: a 3-year analysis. Histopathology..

[166] Muehlenbachs A, de la Rosa VO, Bausch DG, Schafer IJ, Paddock CD, Nyakio JP (2017). Ebola Virus Disease in Pregnancy: Clinical, Histopathologic, and Immunohistochemical Findings. J Infect Dis..

[167] Zaki SR, Shieh W-J, Greer PW, Goldsmith CS, Ferebee T, Katshitshi J (1999). A Novel Immunohistochemical Assay for the Detection of Ebola Virus in Skin: Implications for Diagnosis, Spread, and Surveillance of Ebola Hemorrhagic Fever. J Infect Dis.

[168] Guarner J, Shieh W-J, Dawson J, Subbarao K, Shaw M, Ferebee T (2000). Immunohistochemical and In Situ Hybridization Studies of Influenza A Virus Infection in Human Lungs. Am J Clin Pathol..

[169] Nakajima N, Sato Y, Katano H, Hasegawa H, Kumasaka T, Hata S (2012). Histopathological and immunohistochemical findings of 20 autopsy cases with 2009 H1N1 virus infection. Mod Pathol..

[170] Voltersvik P, Aqrawi LA, Dudman S, Hungnes O, Bostad L, Brokstad KA (2016). Pulmonary changes in Norwegian fatal cases of pandemic influenza H1N1 (2009) infection: a morphologic and molecular genetic study. Influenza Other Respir Viruses..

[171] Ng DL, Al Hosani F, Keating MK, Gerber SI, Jones TL, Metcalfe MG (2016). Clinicopathologic, Immunohistochemical, and Ultrastructural Findings of a Fatal Case of Middle East Respiratory Syndrome Coronavirus Infection in the United Arab Emirates, April 2014. Am J Pathol..

[172] Dettmeyer R, Baasner A, Schlamann M, Haag C, Madea B (2002). Coxsackie B3 Myocarditis in 4 Cases of Suspected Sudden Infant Death Syndrome: Diagnosis by Immunohistochemical and Molecular-Pathologic Investigations. Pathol Res Prac..

[173] Gaaloul I, Riabi S, Harrath R, Evans M, Salem NH, Mlayeh S (2012). Sudden unexpected death related to enterovirus myocarditis: histopathology, immunohistochemistry and molecular pathology diagnosis at post-mortem. BMC Infect Dis..

[174] Gao L, Lin P, Liu S, Lei B, Chen Q, Yu S (2014). Pathological examinations of an enterovirus 71 infection: an autopsy case. Int J Clin Exp Pathol..

[175] Zhang H, Li Y, McClean DR, Richardson PJ, Florio R, Sheppard M (2004). Detection of enterovirus capsid protein VP1 in myocardium from cases of myocarditis or dilated cardiomyopathy by immunohistochemistry: further evidence of enterovirus persistence in myocytes. Med Microbiol Immunol..

[176] Zhang H, Li Y, Peng T, Aasa M, Zhang L, Yang Y (2000). Localization of Enteroviral Antigen in Myocardium and Other Tissues from Patients with Heart Muscle Disease by an Improved Immunohistochemical Technique. J Histochem Cytochem..

[177] Blight K, Rowland R, Hall PD, Lesniewski RR, Trowbridge R, LaBrooy JT (1993). Immunohistochemical detection of the NS4 antigen of hepatitis C virus and its relation to histopathology. Am J Pathol..

[178] Nayak NC, Sathar SA (1999). Immunohistochemical detection of hepatitis C virus antigen in paraffin embedded liver biopsies from patients with chronic liver disease. Acta Histochemica..

[179] Verslype C, Nevens F, Sinelli N, Clarysse C, Pirenne J, Depla E (2003). Hepatic immunohistochemical staining with a monoclonal antibody against HCV-E2 to evaluate antiviral therapy and reinfection of liver grafts in hepatitis C viral infection. J Hepatol..

[180] De Brito T, Siqueira SAC, Santos RTM, Nassar ES, Coimbra TLM, Alves VAF (1992). Human fatal yellow fever. Pathol Res Pract..

[181] Quaresma JAS, Barros VLRS, Pagliari C, Fernandes ER, Andrade HF, Vasconcelos PFC (2007). Hepatocyte lesions and cellular immune response in yellow fever infection. Trans R Soc Trop Med Hyg..

[182] Gupta P, Jagya N, Pabhu SB, Durgapal H, Acharya SK, Panda SK (2012). Immunohistochemistry for the diagnosis of hepatitis E virus infection: HEV detection in archival specimens. J Viral Hepat..

[183] Bartaquini RT, Torquato RB, Fernandes ER, Guedes F, de Castro BS, Katz ISS (2020). Evaluation of polyclonal anti-RNP IgG antibody for rabies diagnosis by indirect rapid immunohistochemistry test. Acta Tropica..

[184] Farahtaj F, Alizadeh L, Gholami A, Tahamtan A, Shirian S, Fazeli M (2019). Natural Infection with Rabies Virus: A Histopathological and Immunohistochemical Study of Human Brains. Osong Public Health Res Perspect..

[185] Jogai S, Radotra B, Banerjee A (2000). Immunohistochemical study of human rabies. Neuropathology..

[186] Shieh W-J, Hsiao C-H, Paddock CD, Guarner J, Goldsmith CS, Tatti K (2005). Immunohistochemical, in situ hybridization, and ultrastructural localization of SARS-associated coronavirus in lung of a fatal case of severe acute respiratory syndrome in Taiwan. Hum Pathol..

[187] Adachi T, Chong J-M, Nakajima N, Sano M, Yamazaki J, Miyamoto I (2020). Clinicopathologic and Immunohistochemical Findings from Autopsy of Patient with COVID-19. Japan. Emerg Infect Dis..

[188] Best Rocha A, Stroberg E, Barton LM, Duval EJ, Mukhopadhyay S, Yarid N (2020). Detection of SARS-CoV-2 in formalin-fixed paraffin-embedded tissue sections using commercially available reagents. Lab Inv..

[189] Colmenero I, Santonja C, Alonso-Riaño M, Noguera-Morel L, Hernández-Martín A, Andina D, et al. SARS-CoV-2 endothelial infection causes COVID-19 chilblains: histopathological, immunohistochemical and ultraestructural study of 7 paediatric cases. Br J Dermatol. 2020; https://www.ncbi.nlm.nih.gov/pmc/articles/PMC7323219/. 10.1111/bjd.19327 PMC7323219 32562567

[190] Martines RB, Ritter JM, Matkovic E, Gary J, Bollweg BC, Bullock H (2020). Pathology and Pathogenesis of SARS-CoV-2 Associated with Fatal Coronavirus Disease. United States. Emerg Infect Dis..

[191] Sauter JL, Baine MK, Butnor KJ, Buonocore DJ, Chang JC, Jungbluth AA, et al. Insights into pathogenesis of fatal COVID-19 pneumonia from histopathology with immunohistochemical and viral RNA studies. Histopathology. 2020; https://www.ncbi.nlm.nih.gov/pmc/articles/PMC7361244/. 10.1111/his.14201 PMC7361244 32614086

[192] Szabolcs M, Sauter JL, Frosina D, Geronimo JA, Hernandez E, Selbs E (2021). Identification of Immunohistochemical Reagents for In Situ Protein Expression Analysis of Coronavirus-associated Changes in Human Tissues. Appl Immunohistochem Mol Morphol..

[193] Cazzato G, Mazzia G, Cimmino A, Colagrande A, Sablone S, Lettini T (2021). SARS-CoV-2 and Skin: The Pathologist’s Point of View. Biomolecules..

[194] Bollweg BC, Silva-Flannery L, Spivey P, Hale GL (2018). Optimization of commercially available Zika virus antibodies for use in a laboratory-developed immunohistochemical assay. J Pathol Clin Res..

[195] Armah HB, Wang G, Omalu BI, Tesh RB, Gyure KA, Chute DJ (2007). Systemic Distribution of West Nile Virus Infection: Postmortem Immunohistochemical Study of Six Cases. Brain Pathol..

[196] Cushing MM, Brat DJ, Mosunjac MI, Hennigar RA, Jernigan DB, Lanciotti R (2004). Fatal West Nile Virus Encephalitis in a Renal Transplant Recipient. Am J Clin Pathol..

[197] Paddock CD, Nicholson WL, Bhatnagar J, Goldsmith CS, Greer PW, Hayes EB (2006). Fatal Hemorrhagic Fever Caused by West Nile Virus in the United States. Clin Infect Dis..

[198] Guarner J, de Leon-Bojorge B, Lopez-Corella E, Ferebee-Harris T, Gooding L, Garnett CT (2003). Intestinal Intussusception Associated With Adenovirus Infection in Mexican Children. Am J Clin Pathol..

[199] Kosulin K, Geiger E, Vécsei A, Huber W-D, Rauch M, Brenner E (2016). Persistence and reactivation of human adenoviruses in the gastrointestinal tract. Clin Microbiol Infect.

[200] Weidner A-S, Panarelli NC, Rennert H, Jessurun J, Yantiss RK (2016). Immunohistochemistry Improves the Detection of Adenovirus in Gastrointestinal Biopsy Specimens From Hematopoietic Stem Cell Transplant Recipients. Am J Clin Pathol..

[201] Kucinskaite-Kodze I, Petraityte-Burneikiene R, Zvirbliene A, Hjelle B, Medina RA, Gedvilaite A (2011). Characterization of monoclonal antibodies against hantavirus nucleocapsid protein and their use for immunohistochemistry on rodent and human samples. Arch Virol..

[202] Latus J, Tenner-Racz K, Racz P, Kitterer D, Cadar D, Ott G, et al. Detection of Puumala Hantavirus Antigen in Human Intestine during Acute Hantavirus Infection. PLoS ONE. 2014;9(5) https://www.ncbi.nlm.nih.gov/pmc/articles/PMC4032337/. 10.1371/journal.pone.0098397 PMC4032337 24857988

[203] Molina-Ruiz AM, Santonja C, Rütten A, Cerroni L, Kutzner H, Requena L (2015). Immunohistochemistry in the Diagnosis of Cutaneous Viral Infections- Part II: Cutaneous Viral Infections by Parvoviruses, Poxviruses, Paramyxoviridae, Picornaviridae. Retroviruses and Filoviruses. Am J Dermatopathol..

[204] Grocott RG (1955). A Stain for Fungi in Tissue Sections and Smears: Using Gomori’s Methenamine-Silver Nitrate Technic. Am J Clin Pathol.

[205] Lamps LW, Talley LR, Nolen CT, Womack J, Scott MA. A Combined Hematoxylin and EosinIMethnamine Silver Stain for the Histological Diagnosis of Fungi in Tissue Sections. J Histotechnol. 2013; https://www.tandfonline.com/doi/abs/10.1179/his.2000.23.4.341.

[206] Schwarz J (1982). The diagnosis of deep mycoses by morphologic methods. Hum Pathol..

[207] Inkomlue R, Larbcharoensub N, Karnsombut P, Lerksuthirat T, Aroonroch R, Lohnoo T (2016). Development of an Anti-Elicitin Antibody-Based Immunohistochemical Assay for Diagnosis of Pythiosis. J Clin Microbiol..

[208] Piao Y-S, Zhang Y, Yang X, He C-Y, Liu H-G (2008). The use of MUC5B antibody in identifying the fungal type of fungal sinusitis. Hum Pathol..

[209] Challa S, Uppin SG, Uppin MS, Pamidimukkala U, Vemu L (2015). Diagnosis of filamentous fungi on tissue sections by immunohistochemistry using anti-aspergillus antibody. Med Mycol..

[210] Choi JK, Mauger J, McGowan KL (2004). Immunohistochemical Detection of *Aspergillus* Species in Pediatric Tissue Samples. Am J Clin Pathol..

[211] Phillips P, Weiner MH (1987). Invasive aspergillosis diagnosed by immunohistochemistry with monoclonal and polyclonal reagents. Hum Pathol..

[212] Marques ME, Coelho KI, Sotto MN, Bacchi CE (1992). Comparison between histochemical and immunohistochemical methods for diagnosis of sporotrichosis. J Clin Pathol..

[213] Monteagudo C, Marcilla A, Mormeneo S, Llombart-Bosch A, Sentandreu R (1995). Specific Immunohistochemical Identification of Candida albicans in Paraffin-embedded Tissue With a New Monoclonal Antibody (1B12). Am J Clin Pathol..

[214] Tsunemi T, Kamata T, Fumimura Y, Watanabe M, Yamawaki M, Saito Y (2001). Immunohistochemical Diagnosis of *Cryptococcus neoformans* var. *gattii* Infection in Chronic Meningoencephalitis: the First Case in Japan. Intern Med..

[215] Amato VS, Tuon FF, de Andrade JHF, Bacha H, Pagliari C, Fernandes ER (2009). Immunohistochemistry and polymerase chain reaction on paraffin-embedded material improve the diagnosis of cutaneous leishmaniasis in the Amazon region. Int J Dermatol..

[216] Sánchez-Romero C, Júnior HM, VLR da M, Freitas LM, C de M S, Mariano FV (2020). Immunohistochemical and Molecular Diagnosis of Mucocutaneous and Mucosal Leishmaniasis. Int J Surg Pathol..

[217] Gonzalez K, Calzada JE, Díaz R, Paz H, García V, Miranda A (2019). Performance of immunohistochemistry as a useful tool for the diagnosis of cutaneous leishmaniasis in Panama. Cent Am. Parasitol Int..

[218] Lunedo SN, Thomaz-Soccol V, de Castro EA, Telles JEQ (2012). Immunocytochemical and immunohistochemical methods as auxiliary techniques for histopathological diagnosis of cutaneous leishmaniasis. Acta Histochemica..

[219] Marques FA, Soares RP, Almeida GG, Souza CC, Melo MN, Pinto SA (2017). Effectiveness of an immunohistochemical protocol for Leishmania detection in different clinical forms of American tegumentary leishmaniasis. Parasitol Int..

[220] Andres TL, Dorman SA, Winn WC, Trainer TD, Perl DP (1981). Immunohistochemical Demonstration of Toxoplasma gondii. Am J Clin Pathol..

[221] Brouland J, Audouin J, Hofman P, Le Tourneau A, Basset D, Rio B (1996). Bone marrow involvement by disseminated toxoplasmosis in acquired immunodeficiency syndrome: The value of bone marrow trephine biopsy and immunohistochemistry for the diagnosis. Hum Pathol..

[222] Pinto-Ferreira F, de Nino B, SL MFDC, Monica TC, Britto IC, Signori A (2020). Isolation, genetic and immunohistochemical identification of Toxoplasma gondii from human placenta in a large toxoplasmosis outbreak in southern Brazil, 2018. Infect Genet Evol.

[223] Barth TFE, Herrmann TS, Tappe D, Stark L, Grüner B, Buttenschoen K, et al. Sensitive and Specific Immunohistochemical Diagnosis of Human Alveolar Echinococcosis with the Monoclonal Antibody Em2G11. PLoS Negl Trop Dis. 2012;6(10) https://www.ncbi.nlm.nih.gov/pmc/articles/PMC3493387/. 10.1371/journal.pntd.0001877 PMC3493387 23145198

[224] Reinehr M, Micheloud C, Grimm F, Kronenberg PA, Grimm J, Beck A (2020). Pathology of Echinococcosis: A Morphologic and Immunohistochemical Study on 138 Specimens With Focus on the Differential Diagnosis Between Cystic and Alveolar Echinococcosis. Am J Surg Pathol..

[225] Genrich GL, Zaki SR, Paddock CD, Greer PW, Barnwell JW, Guarner J (2007). Fatal malaria infection in travelers: Novel immunochemical assays for the detection of *Plamodium falciparum* in tissues and implications for pathogenesis. Am J Trop Med Hyg..

[226] Azevedo PHR, Xavier MAP, da Silva GN, da Costa PA, Carneiro CM, Brasileiro Filho G (2018). Anti-serum validation for use in immunohistochemistry for Trypanosoma cruzi detection. Revista da Sociedade Brasileira de Medicina Tropical..

[227] Ferreira-Filho JCR, Braz LMA, Andrino MLA, Yamamoto L, Kanashiro EHY, da Silva AMG (2021). A sensitive and reliable quantitative immunohistochemistry technique to evaluate the percentage of Trypanosoma cruzi-infected tissue area. Parasitol Int..

[228] Rauch J, Muntau B, Eggert P, Tappe D (2018). Rickettsia typhi as Cause of Fatal Encephalitic Typhus in Hospitalized Patients, Hamburg, Germany, 1940–1944. Emerg Infect Dis..

[229] Oumarou Hama H, Barbieri R, Guirou J, Chenal T, Mayer A, Ardagna Y (2020). An outbreak of relapsing fever unmasked by microbial paleoserology, 16th century. France. Am J Phys Anthropol..

[230] Barbieri R, Mekni R, Levasseur A, Chabrière E, Signoli M, Tzortzis S (2017). Paleoproteomics of the Dental Pulp: The plague paradigm. PLOS ONE..

