# Supplementary material for: Immunohistochemical diagnosis of human infectious diseases: a review
Source: Diagn Pathol. 2022 Jan 30;17:17. doi: 10.1186/s13000-022-01197-5 (PMC8801197; doi:10.1186/s13000-022-01197-5)
Supplement: Supplementary file 1 — Additional file 1: Supplementary Material [file 13000_2022_1197_MOESM1_ESM.docx]

**Supplementary Material**

**1.** Deparaffinise the sections with xylem twice for 5 minutes and rehydrate with graduated concentrations of ethanol (absolute, 95% and 70%) for 5 minutes per series, then with distilled water for 5 minutes.

**2.** Antigen retrieval by heating tissue sections or by the digestion of proteases, if necessary.

**3.** Block endogenous enzymatic activities by immersing the slides in 0.5% methanol solution for 30 minutes.

**4.** Block non-specific staining by incubating tissue sections with non-immune serum for 10 minutes.

**5.** Incubate the sections with the primary antiserum for 30-60 minutes at room temperature and wash the sections three times with buffer for 5 minutes.

**6.** Apply a second biotinylated antibody to immunoglobulin and incubate for 30-60 minutes at room temperature. Wash the sections three times in buffer for 5 minutes.

**7.** Apply an avidin-biotin (or streptavidin-biotin) peroxidase (or alkaline phosphatase) reagent prepared according to the manufacturer instructions. Incubate for 30 minutes at room temperature and wash the sections three times in buffer for 5 minutes.

**8.** Reveal the immune reaction by applying a substrate chromogen solution to sections such as 3.3'-diaminobenzidine (DAB) for 1-5 min and wash with deionised water for 5 minutes.

**9.** Counterstain the slides with hematoxylin before dehydrating and covering.

**Figure S:** Immunostaining procedures
